# Methylprednisolone Plasma Concentrations During Cardiac Surgery With Cardiopulmonary Bypass in Pediatric Patients

**DOI:** 10.3389/fcvm.2021.640543

**Published:** 2021-08-25

**Authors:** Annewil van Saet, Gerdien A. Zeilmaker-Roest, Kevin M. Veen, Saskia N. de Wildt, Fritz Sorgel, Robert J. Stolker, Ad J. J. C. Bogers, Dick Tibboel

**Affiliations:** ^1^Department of Anesthesiology, Erasmus Medical Center, Rotterdam, Netherlands; ^2^Department of Intensive Care and Pediatric Surgery, Erasmus Medical Center, Rotterdam, Netherlands; ^3^Department of Cardiothoracic Surgery, Erasmus Medical Center, Rotterdam, Netherlands; ^4^Department of Pharmacology and Toxicology, Radboud Institute for Health Sciences, Nijmegen, Netherlands; ^5^Faculty of Medicine, Institute of Pharmacology, University Duisburg-Essen, Essen, Germany; ^6^Department of Clinical Pharmacology, Institute for Biomedical and Pharmaceutical Research, Nürnberg-Heroldsberg, Germany

**Keywords:** methyprednisolone, pediatric, cardiopulmonary bypass, cardiac surgery, *in vivo*

## Abstract

**Introduction:** To our knowledge, methylprednisolone pharmacokinetics and plasma concentrations have not been comprehensively investigated in children with congenital heart disease undergoing cardiac surgery with cardiopulmonary bypass. It is unknown whether there is a significant influence of cardiopulmonary bypass on the plasma concentrations of methylprednisolone and whether this may be an explanation for the limited reported efficacy of steroid administration in cardiac surgery with cardiopulmonary bypass.

**Methods:** The study was registered in the Dutch Trial Register (NTR3579; https://www.trialregister.nl/trial/3428). Methylprednisolone 30 mg/kg was administered as an intravenous bolus after induction of anesthesia. Methylprednisolone concentration was measured with liquid chromatography tandem mass spectrometry and analyzed using linear mixed-effects modeling.

**Results:** Thirty-nine patients were included in the study, of which three were excluded. There was an acute decrease in observed methylprednisolone plasma concentration on initiation of cardiopulmonary bypass (median = 26.8%, range = 13.9–48.14%, *p* < 0.001). We found a lower intercept (*p* = 0.02), as well as a less steep slope of the model predicted methylprednisolone concentration vs. time curve for neonates (*p* = 0.048). A lower intercept (*p* = 0.01) and a less steep slope (p = 0.0024) if the volume of cell saver blood processed was larger than 91 ml/kg were also found.

**Discussion:** We report similar methylprednisolone plasma concentrations as earlier studies performed in children undergoing cardiopulmonary bypass, and we confirmed the large interindividual variability in achieved methylprednisolone plasma concentrations with weight-based methylprednisolone administration. A larger volume of distribution and a lower clearance of methylprednisolone for neonates were suggested. The half-life of methylprednisolone in our study was calculated to be longer than 6 h for neonates, 4.7 h for infants, 3.6 h for preschool children and 4.7 h for school children. The possible influence of treatment of pulmonary hypertension with sildenafil and temperature needs to be investigated further.

## Introduction

Every year, thousands of pediatric patients undergo cardiac surgery facilitated by cardiopulmonary bypass (CPB). The use of CPB has profound effects on distribution and elimination of drugs due to hemodilution, changes in protein binding due to the composition of the priming fluid, changes in oxygen delivery to drug-eliminating organs, hypothermia, changes in acid–base status, exclusion of the lungs from the circulation, and uptake of drugs by CPB-circuit components (1–7).

CPB is known to initiate an intense systemic humoral and cellular inflammatory reaction (8), which may lead to multiple organ failure and prolonged stay at the intensive care unit (ICU) (9). To attenuate this inflammatory response, corticosteroids are often administered. There appears to be a limited influence of corticosteroid administration on clinical outcomes in pediatric cardiac surgery (10–12). It is unknown whether the inefficacy of corticosteroid administration in cardiac surgery with CPB is caused (partly) by the effects of CPB on the plasma concentration of steroids.

Even though methylprednisolone (MP) is used often in pediatric CPB patients, few studies are available concerning its pharmacokinetics in pediatric patients (13). One study is available on the effect of CPB on pharmacokinetic (PK) parameters in adults (14). Studies in the adult population cannot automatically be translated to the pediatric cardiac population because of developmental and disease-specific differences in pharmacokinetics and drug effect in children. The goal of the current study was to describe total MP plasma concentrations during CPB in pediatric patients after a single intravenous bolus dose of 30 mg/kg at the induction of anesthesia.

## Methods

This study was performed at the Department of Cardiothoracic Surgery and the Pediatric ICU of the Erasmus Medical Center, Rotterdam, the Netherlands, and was part of a larger single-center observational PK study in children during cardiac surgery requiring CPB. The goal of the study is to measure concentrations of drugs commonly used during pediatric cardiac surgery, with the ultimate goal of performing population PK modeling of these drugs. The research protocol was approved by the institutional Medical Ethical Review Board (MEC2011-400) and registered in the Dutch Trial Register (NTR3579; https://www.trialregister.nl/trial/3428). According to Dutch law, preoperative written informed consent was obtained from the patient's parents or legal guardians and in children aged 12 to 18 years from the patient as well.

Patients were included if they were younger than 18 years and undergoing elective (congenital) cardiac surgery requiring CPB. Exclusion criteria were no informed consent, no use of CPB planned, and no MP received.

For every patient, we collected the following covariates: age group (neonates 0–30 days of age, infants 30 days to 1 year of age, preschool children 1–4 years of age, school children 4–12 years of age), sex, weight, body surface area (BSA), preoperative treatment with diuretics for volume overload (yes or no), preoperative treatment with sildenafil for pulmonary hypertension (PHT; yes or no), presence of cyanosis before and/or after surgery (yes or no), and STSEACTS mortality risk category (15). We collected the following CPB management covariates: use of a roller pump vs. a centrifugal pump, oxygenator type used (Table 1), type of arteriovenous tubing used (Table 1), volume of priming fluid, red blood cells in the priming fluid (yes or no), fresh frozen plasma in the priming fluid (yes or no), duration of CPB, duration of aorta occlusion, use of a hemofilter (yes or no), and whether there were multiple runs on CPB (yes or no). We collected the following surgery-related covariates: minimum temperature during CPB (normothermia >35°C, mild hypothermia: 32–35°C, moderate hypothermia: 28–32°C, severe hypothermia 20–28°C, deep hypothermia: <20°C), blood loss during surgery, diuresis during surgery, the volume of cell saver blood processed, and the volume of cell saver blood returned to the patient.

**Table 1:** CPB systems.

	**Oxygenator**	**Reservoir**	**Arterial filter**	**Venous filter cardiotomy**	**Defoaming sponge**	**Silicone tubing**	**PVC tubing**	**Priming volume**
Neonatal roller	Capiox® FX05, Terumo Europe NV, Leuven, Belgium, Hollow fiber, Polycarbonate housing, polypropylene membrane 0.5 m^2^, priming volume 43ml, X-coating™	Open hardshell polycarbonate, minimum capacity 15 ml, maximum capacity 1,000 ml	Integrated polyester screen type, Surface area 130 cm^2^, pore size 32μm	Polyester screen type, pore size 47μm	Polyurethane	Sorin® Kids neonate set, custom made, Sorin Group, Mirandola, Italy, 0.02 m^2^ contact surface area, Phisio coating	Sorin® Kids neonate set, custom made, Sorin Group, Mirandola, Italy, 0.07 m^2^ contact surface area, Phisio coating	230 ml
Neonatal Centrifugal, BP-50 Bio-Pump®, Medtronic, Minneapolis, MN, USA, Pump casing polycarbonate, priming volume 48 ml	Capiox® FX05, Terumo Europe NV, Leuven, Belgium, Polycarbonate housing, polypropylene membrane 0.5 m^2^, priming volume 43 ml, X-coating™	Open hardshell polycarbonate, minimum capacity 15 ml, maximum capacity 1,000 ml	Integrated polyester screen type, Surface area 130 cm^2^, pore size 32μm	Polyester screen type, pore size 47μm	Polyurethane	Sorin® Kids neonate set, custom made, Sorin Group, Mirandola, Italy, 0.02 m^2^ contact surface area, Phisio coating	Sorin® Kids neonate set, custom made, Sorin Group, Mirandola, Italy, 0.07 m^2^ contact surface area, Phisio coating	230 ml
Infant Roller	Sorin Kids D101, Sorin Group, Mirandola, Italy, Hollow fiber, Polycarbonate housing, polypropylene membrane 0.61 m^2^, priming volume 87 ml, Phisio coating	Open hardshell, polycarbonate, minimum capacity 30 ml, maximum capacity 1,500 ml	Sorin Kids D131 stand-alone arterial filter, Sorin Group, Mirandola, Italy, Polycarbonate housing, phosphorylchloride screen type membrane, Surface area 27 cm^2^, pore size 40μm, priming volume 28 ml	Polyester, pore size 51μm	Polyurethane	Sorin® Kids, custom made, Sorin Group, Mirandola, Italy 0.02, m^2^ contact surface area, Phisio coating	Sorin® Kids custom made, Sorin Group, Mirandola, Italy, 0.08 m^2^ contact surface area, Phisio coating	420 ml
Pediatric Centrifugal, Revolution, Sorin Group, Mirandola, Italy. Pump casing polycarbonate priming volume 57 ml	Capiox® FX15, Terumo Europe NV, Leuven, Belgium, Hollow fiber, Polycarbonate housing, polypropylene membrane 1.5 m^2^, priming volume 144 ml, X-coating™	Open hardshell polycarbonate, minimum capacity 70 or 200 ml, maximum capacity 3,000 or 4,000 ml	Integrated polyester screen type, Surface area 360 cm^2^, pore size 32μm	Polyester screen type, pore size 47μm	Polyurethane	None	Sorin® Kids Pediatric set, custom made, Sorin Group, Mirandola, Italy, 0.11 m^2^ contact surface area, Phisio coating	700 ml

### Anesthesia

Anesthesia was induced with an inhalational (sevoflurane) or intravenous (propofol and/or midazolam and sufentanil) anesthetic technique. After placement of an intravenous cannula, balanced anesthesia was performed with midazolam, sufentanil, and pancuronium. After induction of anesthesia, patients received MP–hemisuccinate (a prodrug of MP; Solumedrol, Pfizer BV, Capelle a/d IJssel, NL) 30 mg/kg with a maximum dose of 2 g as an intravenous bolus at the discretion of the attending anesthesiologist. No preoperative dose of MP was given, and MP was not added to the prime fluid. Anesthesia was maintained with a midazolam continuous infusion and sufentanil as a continuous infusion or as bolus doses at the discretion of the attending anesthesiologist. In children older than 1 year, anesthesia was alternatively maintained with a continuous infusion of propofol and remifentanil.

### CPB

The CPB systems used are described in Table 1. Priming fluid contained fresh frozen plasma and Gelofusine® (B. Braun Medical BV, Melsungen, Germany). Red blood cells were added to the priming to achieve a hematocrit of 28% during CPB. The priming fluid was completed with 0.5 g/kg mannitol, 0.5 g/kg human albumin (Cealb®, Sanquin, Amsterdam, the Netherlands), 4.2 IU heparin per ml of priming volume (Leo Pharma BV, Amsterdam, the Netherlands), and 2 to 5 ml sodium bicarbonate 8.4% (B. Braun Medical BV, Melsungen, Germany). Anticoagulation was established with an initial bolus of 300 IU/kg of porcine heparin with additional heparin administered to maintain an activated clotting time higher than 480 s during the entire procedure. During CPB, non-pulsatile blood flow was administered with flow rates between 1.8 and 3.2 L/min per m^2^ BSA. Alpha-stat pH-management was used. When deemed necessary, conventional ultrafiltration was applied during CPB. According to the institutional protocol, no modified ultrafiltration was used. The heart was arrested with antegrade St. Thomas Hospital cardioplegic solution 10 to 15 ml/kg with a temperature of 4°C. In most procedures, mild hypothermia (28–32°C) was applied, with the exception of deep hypothermic circulatory arrest (DHCA) procedures (18°C) and beating-heart procedures (36°C).

All blood loss from the moment of skin incision to initiation of CPB and from administration of protamine to skin closure was salvaged together with the residual volume of the CPB circuit in an Electa cell-saving device (Dideco, Sorin Group, Modena, Italy).

Information on hemodynamics, mechanical ventilation, administered medication, and perfusion data was recorded in the automatic anesthesia registration system during the operation and in PICIS (www.PICIS.com) in the ICU.

### Blood Sampling for MP Analyses

Blood samples were taken in 2 ml ethylenediaminetetraacetic acid (EDTA) tubes (3.6 mg EDTA, BD Vacutainer®, BD Life Sciences, Plymouth, UK) from the arterial line. Care was taken not to exceed 5% of total circulating volume including the priming volume of the CPB system. In case of arterial line dysfunction or removal, sampling was stopped. Samples were taken at various random timepoints during the operation at the discretion of the attending anesthesiologist, with emphasis on the following timepoints: after placement of the arterial line, before initiation of CPB, just after reaching full flow on CPB, after reaching the target temperature on CPB, and after weaning from CPB. Samples were stored at 4°C until processing. After centrifugation (10 min at 3,600 rpm), the supernatant serum was transferred to polypropylene cryogenic vials with polypropylene screw caps (Sarstedt Aktiengesellschaft and Co, Nümbrecht, Germany) and stored at −80°C until it was analyzed.

### Assay Method

Drug concentrations were measured using liquid chromatography tandem mass spectrometry (16). The method for MP in human plasma was accurate (bias within −1.7 to 3.9%) and precise (within-run and between-run coefficients of variation below 9.1% in all cases). The mean assay variance of 51 samples evaluated for incurred samples reanalysis in human plasma for MP was 4.8%. No sample value beyond ±20% deviation from the mean was observed.

### Statistical Analysis

Differences between groups were calculated with Mann–Whitney *U* test for numeric data with a non-normal distribution, Fisher exact test for binary categorical data, and χ^2^ test for nominal or ordinal categorical data. Correlations were calculated with Spearman correlations for data with a non-normal distribution and Pearson correlations for data with a normal distribution.

Patient circulating volume was calculated using the following formula:

$$\begin{array}{l} CV\;\left(ml\right)=\;weight\;\left(kg\right)*80 \end{array}$$

To correct for the correlation of repeated measurements in individual patients, MP plasma concentrations after initiation of CPB were analyzed using linear mixed-effects modeling with interindividual variability on intercepts and time slope (baseline model). Based on individual MP concentration vs. time curves, we assumed no changes in PK parameters after weaning from CPB. Covariates were added individually as a fixed effect with an interaction effect between time and the covariate in question, because we expected a different time course for MP plasma concentration after initiation of CPB for covariates. Model assumptions were checked according to the protocol developed by Zuur et al. (17) and were valid. Model fit was assessed by Akaike Information Criterion and Bayesian Information Criterion based on maximum likelihood.

We calculated which percentage of the decrease in MP plasma concentration on initiation of CPB was caused by hemodilution alone or metabolism alone. All calculations are based on a one-compartment model for MP with first-order elimination (13).

The percentage decrease of MP plasma concentration on initiation of CPB based on hemodilution alone was calculated:

$$\begin{array}{l} Amount\;MP=C\left(0\right)*CV \end{array}$$

where amount MP is the amount of MP present in the central compartment before initiation of CPB (mg), *C* (0) is the concentration of MP in the central compartment before initiation of CPB (μg/ml), and CV is circulating volume (ml/kg).

$$\begin{array}{l} \left[MP\right]\;hemodilution=\frac{Amount\;MP}{CV+PV} \end{array}$$

where [MP] hemodilution is the calculated MP plasma concentration after initiation of CPB based on hemodilution alone (μg/ml), CV is patient circulating volume, and PV is priming volume (ml).

$$\begin{array}{l} \%\;decrease\;MP\;hemodilution\\ \;=\left(\frac{C\left(0\right)-\left[MP\right]\;hemodilution}{C\left(0\right)}\right)*100 \end{array}$$

where % decrease MP hemodilution is the % decrease in MP plasma concentration on initiation of CPB based on hemodilution alone.

The percentage decrease of MP plasma concentration on initiation of CPB based on metabolism alone was calculated as follows:

$$\begin{array}{l} C\left(t\right)=C\left(0\right)*\;0.5^{\frac{t}{half-life}} \end{array}$$

where *C* (*t*) is MP plasma concentration in the central compartment after the initiation of CPB (μg/ml), *t* is the amount of time between the MP plasma measurements before and after the initiation of CPB (min), and half-life is MP half-life and equals 138 min (3, 18).

$$\begin{array}{l} \%\;decrease\;\left[MP\right]\;metabolism=\left(\frac{C\left(0\right)-C\left(t\right)}{C\left(0\right)}\right)*100 \end{array}$$

Statistical analyses were performed using R (R Core Team; A Language and Environment for Statistical Computing, R Foundation for Statistical Computing, Vienna, Austria; 2019. www.R-project.org), using the package lme4 (19).

## Results

In a 3.5-year period, 150 patients were included in the larger single-center PK study. MP plasma concentrations were measured in 39 patients. Three patients were excluded: two had missing clinical data, and one received two doses of MP. This left 36 patients for evaluation. A median of 7 (range = 6–10) intraoperative blood samples were taken per patient (total 268 samples). Six patients also had 1 to 4 postoperative samples taken (total 14 samples). Patient and surgery demographics are described in Tables 2, 3.

**Table 2:** Patient demographics.

	**Age (months, median, range)**	**Weight (kg, median, range)**	**BSA (median, range)**	**Gender (M/F, *n*)**	**Cyanosis (*n*)**	**Preoperative treatment with diuretics (*n*)**	**Preoperative treatment for PHT with sildenafil (*n*)**	**STSEACTS mortality category (median, range)**
All (*n* = 36)	7.2 (0.2–117.2)	7.8 (3–33.5)	40 (21–117)	22/14	12	20	2	2 (1–5)
Neonates (*n* = 5)	0.36 (0.2–0.5)	3.2 (3–3.6)	22 (21–23)	4/1	2	3	1	3 (2–4)
Infants (*n* = 17)	3.7 (1.3–11.2)	5.9 (3.4–9.7)	34 (23–47)	10/7	6	12	0	2 (1–5)
Preschool (*n* = 7)	28.4 (23.6–36.8)	10.7 (10–15)	51 (49–62)	5/2	4	4	1	2 (2–4)
School (*n* = 7)	57.6 (48.5–117.2)	17.7 (12.7–33.5)	74 (60–117)	3/4	0	1	0	2 (1–4)

*BSA, body surface area; F, female; M, male*.

**Table 3:** Operation demographics.

		**Oxygenator**	**Pump-type roller**	**Priming volume % circulating volume**	**Perfusion time (min; median, range)**	**Cross-clamp time (min; median, range)**	**Min *T* (median, range)**	**SCP (*n*)**	**UF (*n*)**	**Priming PC 0 (*n*)**	**Priming FFP 0 (*n*)**
All (*n* = 36)			30	48.5 (23.1–133)	147 (41–319)	90.5 (0–177) 0 = 2	32 (19–36)	4	29	10	6
Arterial (*n* = 5)	Switch 3 DSAS 1 Aorta 1	Fx05	3	88.5 (82.7–133)	216 (185–280)	134 (104–177)	32 (20–32)	2	5	0	0
Infants (*n* = 17)	VSD 5 AVSD 4 TOF 4 PCPC 1 aorta 2	Fx05	15	52.6 (36.4–108.7)	146 (41–319)	80 (0–167)	32 (19–36)	2	14	4	4
Preschool (*n* = 7)	Mitral valve 2 TOF 1 PCPC 1 TCPC 2	Fx05 2 D101 5	7	39.1 (26.9–53.1)	156 (92–196)	86 (47–127)	31 (30–32)	0	6	4	0
School (*n* = 7)	VSD 3 AVSD 1 RVOT 2 LVOT 1	D101 5 Fx15 2	5	30.7 (23.1–53.3)	103 (46–217)	59 (0–111)	32 (28–34)	0	4	2	1

*AVSD, atrioventricular septum defect; DSAS, dynamic subaortic stenosis; FFP, fresh frozen plasma; Ht, hematocrit; LVOT, left ventricular outflow tract; PCPC, partial cavopulmonary connection; PC, packed red blood cells; RVOT, right ventricular outflow tract; SCP, selective cerebral perfusion; T, temperature; TCPC, total cavopulmonary connection; TOF, tetralogy of Fallot; UF, ultrafiltration; VSD, ventricular septum defect*.

**Figure 1:**
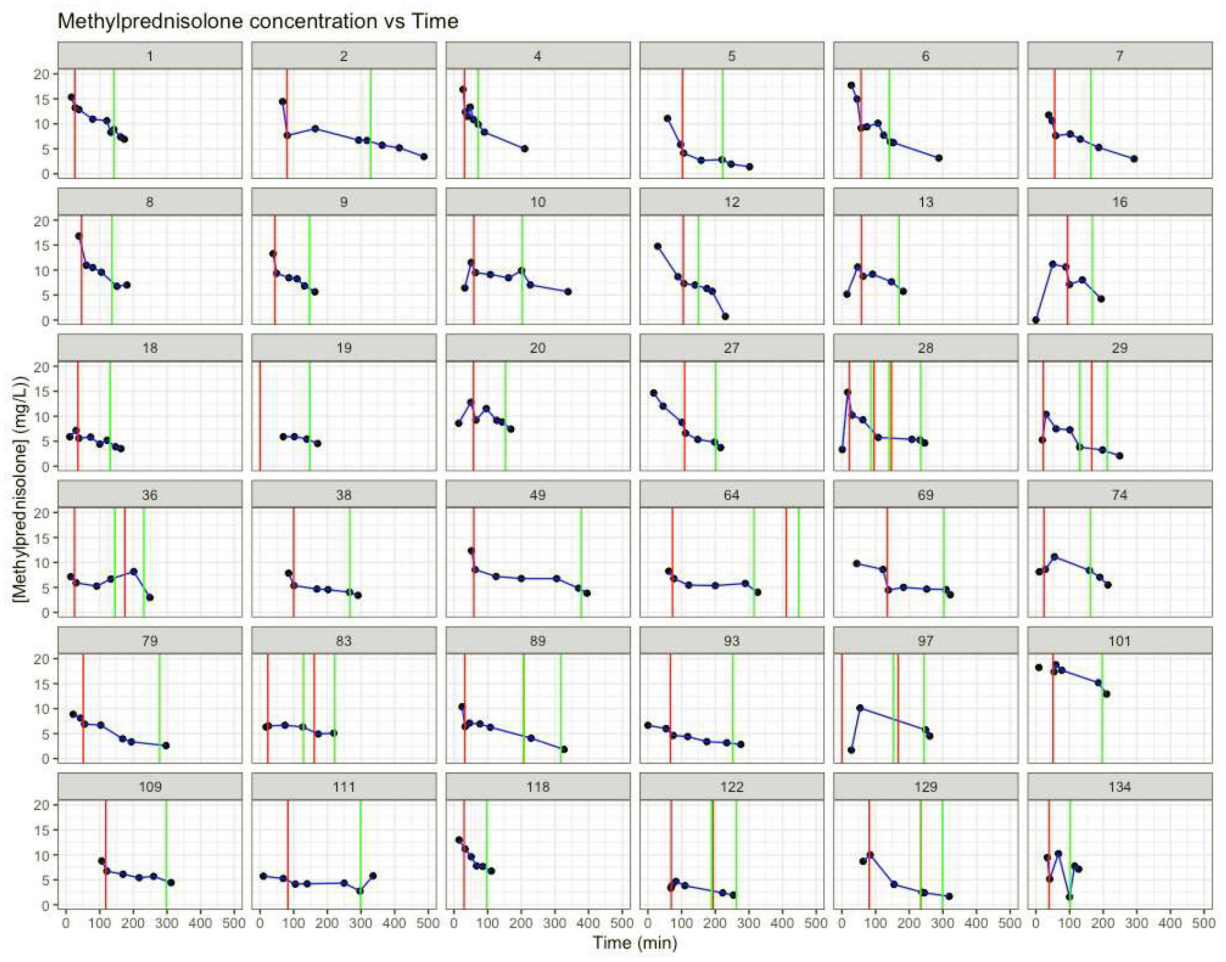
Individual MP concentration (mg/L) vs. time (min). Red vertical lines: time start cardiopulmonary bypass. Green vertical lines: time stop cardiopulmonary bypass. *T* = 0: time of methylprednisolone dosing.

Figure 1 shows the individual patient's MP plasma concentration vs. time plots based on the measured MP plasma concentrations.

There were significant correlations for age group with weight (Spearman ρ = 0.93, *p* < 0.001) and BSA (Spearman ρ = 0.93, *p* < 0.001), oxygenator type (Spearman ρ = 0.84, *p* < 0.001), AV-tubing type (Spearman ρ = 0.84, *p* < 0.001), and volume of priming fluid (Spearman ρ = 0.58, *p* = 0.0002). These correlations are given by the circumstance that we use specific oxygenator types and AV-tubing types in specific age groups.

In the CPB covariates, there were significant correlations for volume of priming fluid and oxygenator type (Spearman ρ = 0.83, *p* < 0.001), volume of priming fluid, and arteriovenous-tubing type (Spearman ρ = 0.83, *p* < 0.001). These correlations are given by the circumstance that specific oxygenators and AV-tubing types have a specific need of priming volume and thus priming type (addition of red blood cells to prevent hemodilution) to prime the CPB system. Perfusion time and aorta occlusion time were also correlated (Spearman ρ = 0.85, *p* < 0.001).

**Figure 2:**
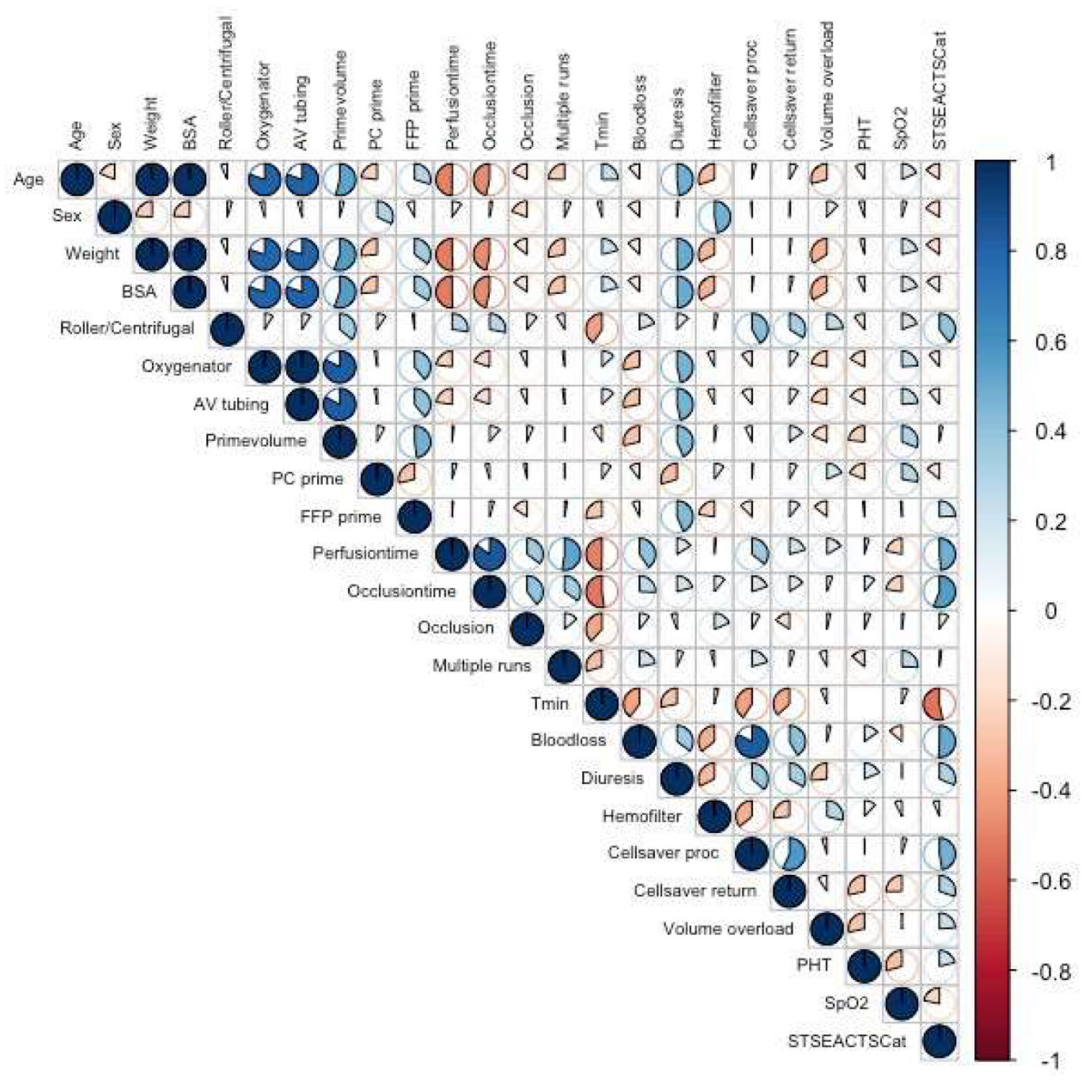
Correlation plot of correlations between covariates calculated with Spearman correlations. AV, arteriovenous; BSA, body surface area; FFP, fresh frozen plasma; PC, red blood cell concentrate; PHT, pulmonary hypertension; proc, processed; return, returned; SpO2 peripheral oxygen saturation; STSEACTSCat, STSEACTS mortality risk category; Tmin, minimum temperature achieved during CPB.

In the surgery covariates, there was a significant correlation for the volume of cell saver blood processed and blood loss (Spearman ρ = 0.82, *p* < 0.001) and a weak but significant correlation for the volume of cell saver blood returned (Spearman ρ = 0.57, *p* = 0.003) (Figure 2).

Compared to other age groups, neonates had a significantly larger volume of priming fluid as a percentage of circulating volume (median = 88.5%, range = 82.7–133% vs. median = 46.2%, range = 23.1–108.7%; *p* < 0.001) and per kilogram of body weight (median = 80 ml/kg, range = 74–120 ml/kg vs. median = 37 ml/kg, range = 19–98 ml/kg; *p* < 0.001). They had a significantly longer perfusion time (median = 216 min, range = 185–280 min vs. median = 138 min, range = 41–319 min; *p* < 0.05) and cross-clamp time (median = 134 min, range = 104–177 min vs. median = 75 min, range = 0–167 min; *p* < 0.05). There was more blood processed in the cell saver per kilogram of body weight (median = 95 ml/kg, range = 60–337 ml/kg vs. 42 ml/kg, range = 11–220 ml/kg; *p* < 0.05), even though there were no significant differences in blood loss, more cell saver blood returned per kilogram of body weight (median = 38 ml/kg, range = 33–168 ml/kg vs. 16 ml/kg, range = 5–143 ml/kg; *p* < 0.05), and a larger volume ultrafiltrated per kilogram of body weight (median = 60 ml/kg, range = 21–99 ml/kg vs. 23 ml/kg, range = 0–68 ml/kg; *p* < 0.05). There were no significant differences for any of the other covariates.

Children who had more cell saver blood processed were younger (median = 39 days, range = 6–340 vs. 502 days, range = 8–3,565 days, *p* < 0.05) and had corresponding lower body weight (median = 3.4 kg, range = 3–9.7, vs. 10 kg, range = 3.6–33.5, *p* < 0.05) and BSA (median = 23, range = 21–47 vs. 49, range = 23–117, *p* < 0.05). Perfusion was more often performed with a centrifugal pump (44.4 vs. 7.4%, *p* = 0.024), and they had a higher incidence of multiple perfusion runs (55.6 vs. 12%, *p* < 0.02). There was a larger blood loss (median = 75 ml/kg, range = 10–259 vs. median = 15.6 ml/kg, range = 1–63, *p* < 0.05), and more cell saver blood returned (median = 64.9 ml/kg, range = 15.9–168.3 vs. median = 14.5 ml/kg, range = 5–50 ml/kg, *p* < 0.05). Priming volume as a percentage of circulating volume (median = 82.7%, range = 45.4%−133% vs. median = 45.8%, range = 23.1–84%, *p* < 0.05) and per kilogram of body weight (median = 74.4 ml/kg, range = 36–120 vs. 36.6 ml/kg, range = 19–76, *p* < 0.05) was larger. Perfusion (median = 216 min, range = 167–319 vs. median = 116 min, range = 41–231, *p* < 0.05) and cross-clamp times (median = 107 min, range = 56–177 vs. median = 75 min, range = 0–167, *p* < 0.05) were longer, and minimum temperature (median = 30°C, range = 19–34°C vs. median = 32°C, range = 28–36°C, *p* < 0.05) was lower. There were no significant differences for any of the other covariates.

We compared the observed MP plasma concentration immediately before and immediately after the initiation of the first run of CPB when available (*n* = 29). The median MP plasma concentration immediately before the initiation of CPB was 10.6 μg/L (range = 5.3–23.3 μg/L). The median MP plasma concentration immediately after the initiation of CPB was 7.3 μg/L (range = 4.1–7.4 μg/L). There was a significant, acute decrease in observed MP plasma concentration with a median of 26.8% (*n* = 29, range = 13.9–48.14%, *p* < 0.001) with a time difference between plasma concentration measurements immediately before and after initiation of CPB of 13 min (median, range = 7–36 min). There was no significant difference in decrease of MP plasma concentration on initiation of CPB between age groups (neonates' median MP plasma concentration immediately before the initiation of CPB 7.1 μg/L (range = 5.3–14.5 μg/L), median MP plasma concentration immediately after the initiation of CPB 5.7 μg/L (range = 4.2–7.7 μg/L), median 22.0%, range = 18.2%−46.9%; infants' median MP plasma concentration immediately before the initiation of CPB 11.9 μg/L (range = 7.1–23.3 μg/L), median MP plasma concentration immediately after the initiation of CPB 9.0 μg/L (range = 4.5–17.4 μg/L), median = 27.3%, range = 13.9–48.1%; preschool median MP plasma concentration immediately before the initiation of CPB 8.3 μg/L (range = 5.9–14.8 μg/L), median MP plasma concentration immediately after the initiation of CPB 6.1 μg/L (range = 4.1–10.2 μg/L), median = 27.2%, range = 22.2–31.5%; school children's median MP plasma concentration immediately before the initiation of CPB 10.6 μg/L (range = 8.7–13.3 μg/L), median MP plasma concentration immediately after the initiation of CPB 7.6 μg/L (range = 5.2–11.2 μg/L), median = 28.0%, range = 14.0–45.6%; all *p* > 0.05). There was no correlation between the percentage decrease in observed MP plasma concentration on initiation of CPB and the absolute volume of priming fluid [Spearman ρ = 0.042 (*p* = 0.83)], priming volume per kilogram of body weight [Spearman ρ = −0.074 [*p* = 0.70)], or priming volume as a percentage of the patient's circulating volume (Spearman ρ = −0.057, *p* = 0.78). Calculation of the expected percentage decrease of MP plasma concentration on initiation of CPB based on hemodilution alone was 31.6% (median, range = 18.8–57.1%), and metabolism alone was 6.4% (median, range = 3.4–16.5%).

### Mixed-Model Analyses

Compared to the baseline linear mixed-effects model, none of the models with covariates included as a fixed effect with an interaction with time showed an improvement in model fit as represented by Akaike information criterion or Bayesian information criterion.

### Patient Covariates

We decided to use age group as a representative proxy variable for weight and BSA, because there were significant correlations between these covariates.

**Figure 3:**
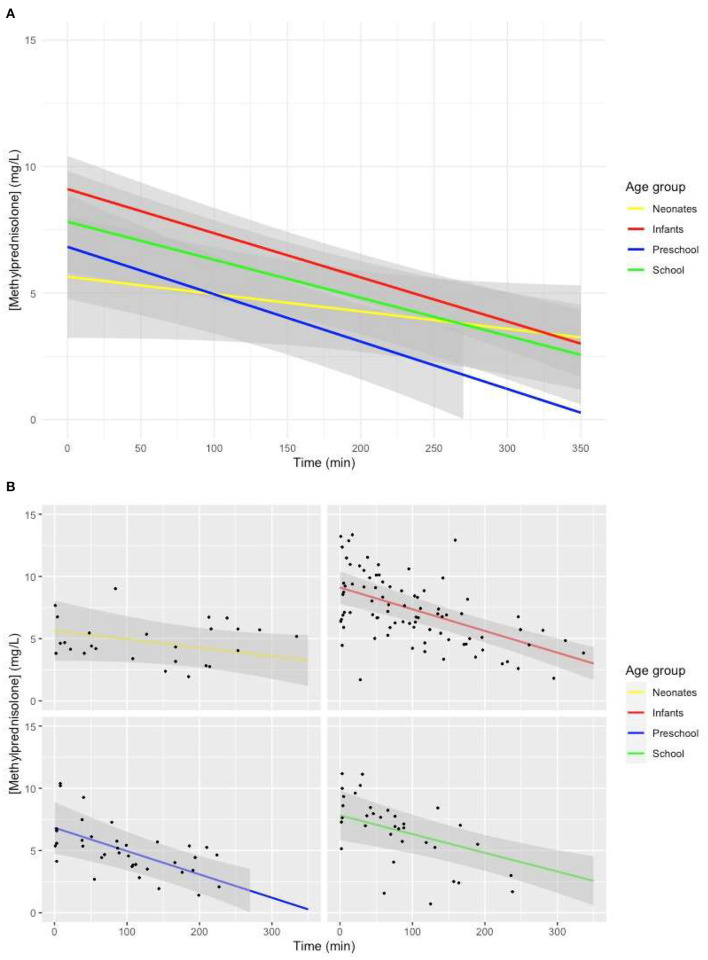
**(A)** Predicted MP concentration (mg/L) vs. time (min) for all age groups after initiation of CPB based on the linear mixed-effects model. Gray ribbons: 95% confidence interval. *T* = 0: start of CPB. **(B)** Predicted MP concentration (mg/L) vs. time (min) for all age groups after initiation of CPB based on the linear mixed-effects model. Gray ribbon: 95% confidence intervals. *T* = 0: start of CPB.

The model with age group added as a fixed effect with an interaction with time showed a lower intercept (*p* = 0.02), as well as a less steep slope for neonates (*p* = 0.048) (Figures 3A,B).

**Figure 4:**
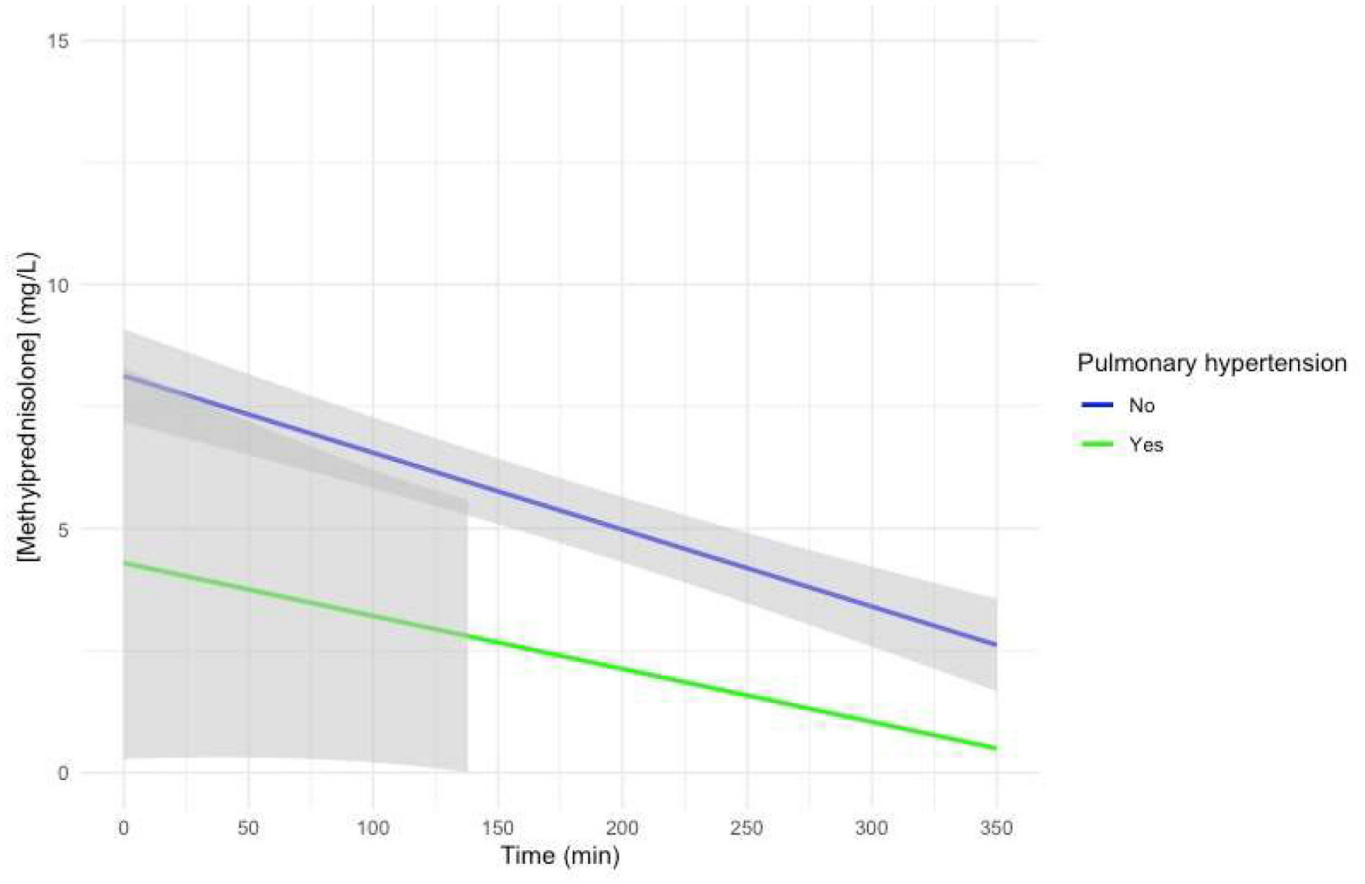
Predicted MP concentration (mg/L) vs. time (min) for patients treated for pulmonary hypertension with sildenafil after initiation of CPB based on the linear mixed-effects model. Gray ribbons: 95% confidence interval. *T* = 0: start of CPB.

Visual representation of the mixed-effects model with preoperative PHT treated with sildenafil added as a fixed effect with an interaction with time showed a non-significantly lower intercept for patients with PHT, with a similar slope, albeit with very large confidence intervals (Figure 4).

Addition of other patient covariates individually to the baseline mixed-effects model showed no significant differences in intercept or slope (Supplementary Figures 1–4).

### CPB Covariates

We decided to not evaluate these covariates separately, but to consider age group as a proxy variable for these factors. We decided to evaluate perfusion time as a categorical variable [perfusion time under 104 min (25% quantile) vs. perfusion time over 195 min (75% quantile)]. Aorta occlusion time was not evaluated separately, as there was a significant correlation with perfusion time.

Addition of CPB covariates individually to the baseline mixed-effects model as a fixed effect with an interaction with time showed no significant differences in intercept or slope (Supplementary Figures 5–11).

### Surgery Covariates

We decided to use the volume of cell saver blood processed as a proxy variable for both blood loss and cell saver blood returned, as there were significant correlations between these covariates. Cell saver blood processed was added as a categorical covariate [cell saver blood processed <26 ml/kg (25% quantile), cell saver blood processed >91 ml/kg (75% quantile)].

**Figure 5:**
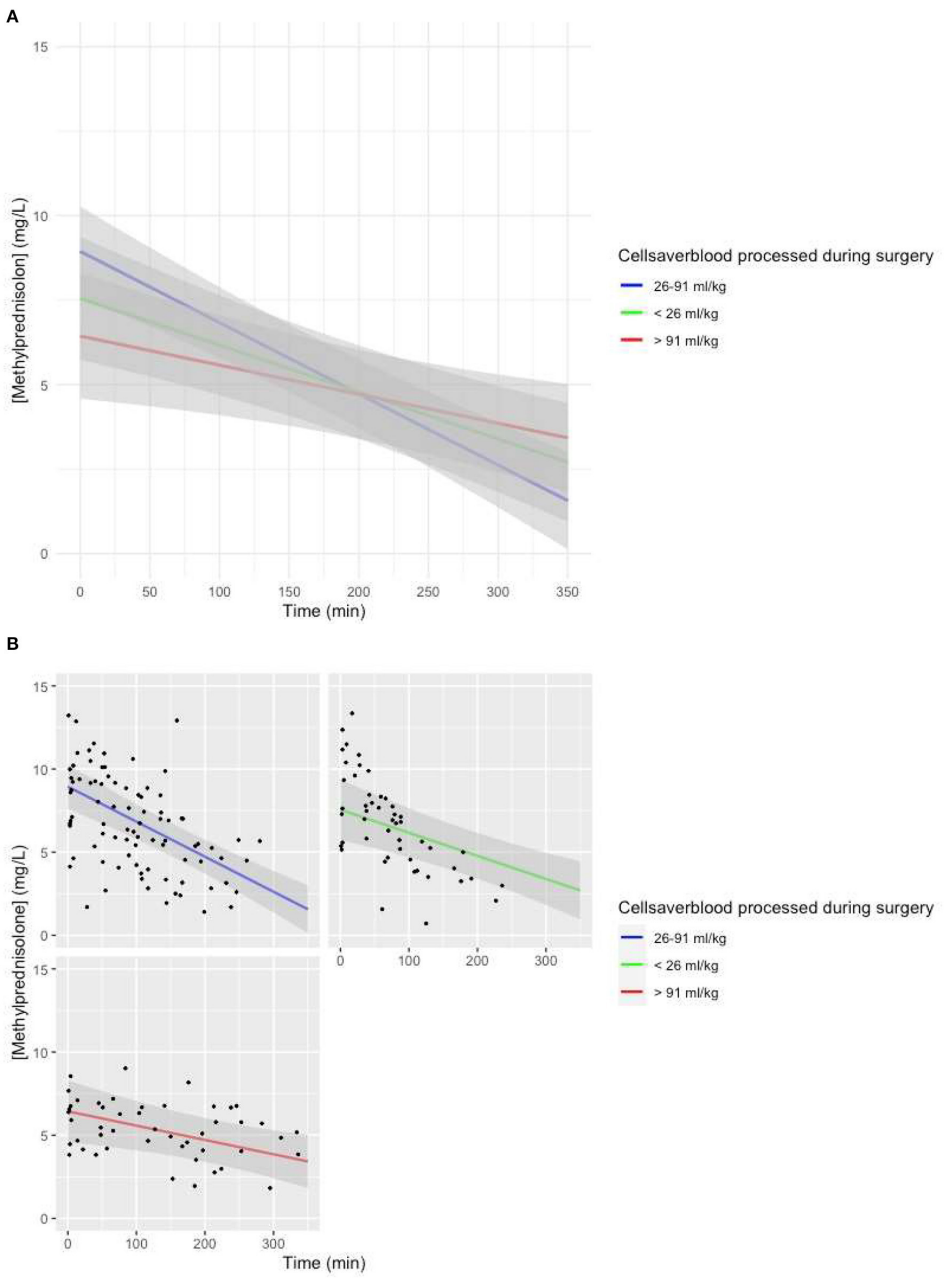
**(A)** Predicted MP concentration (mg/L) vs. time (min) for the volume of cell saver blood processed based on the linear mixed-effects model. Gray ribbon: 95% confidence interval. *T* = 0: start of CPB. **(B)** Predicted MP concentration (mg/L) vs. time (min) for the volume of cell saver blood processed based on the linear mixed-effects model. Gray ribbon: 95% confidence interval. *T* = 0: start of CPB.

The model with the volume of cell saver blood processed added as a fixed effect with an interaction with time showed a lower intercept (*p* = 0.01), as well as a less steep slope (*p* = 0.0024) if the volume of cell saver blood processed was larger than 91 ml/kg (Figures 5A,B).

**Figure 6:**
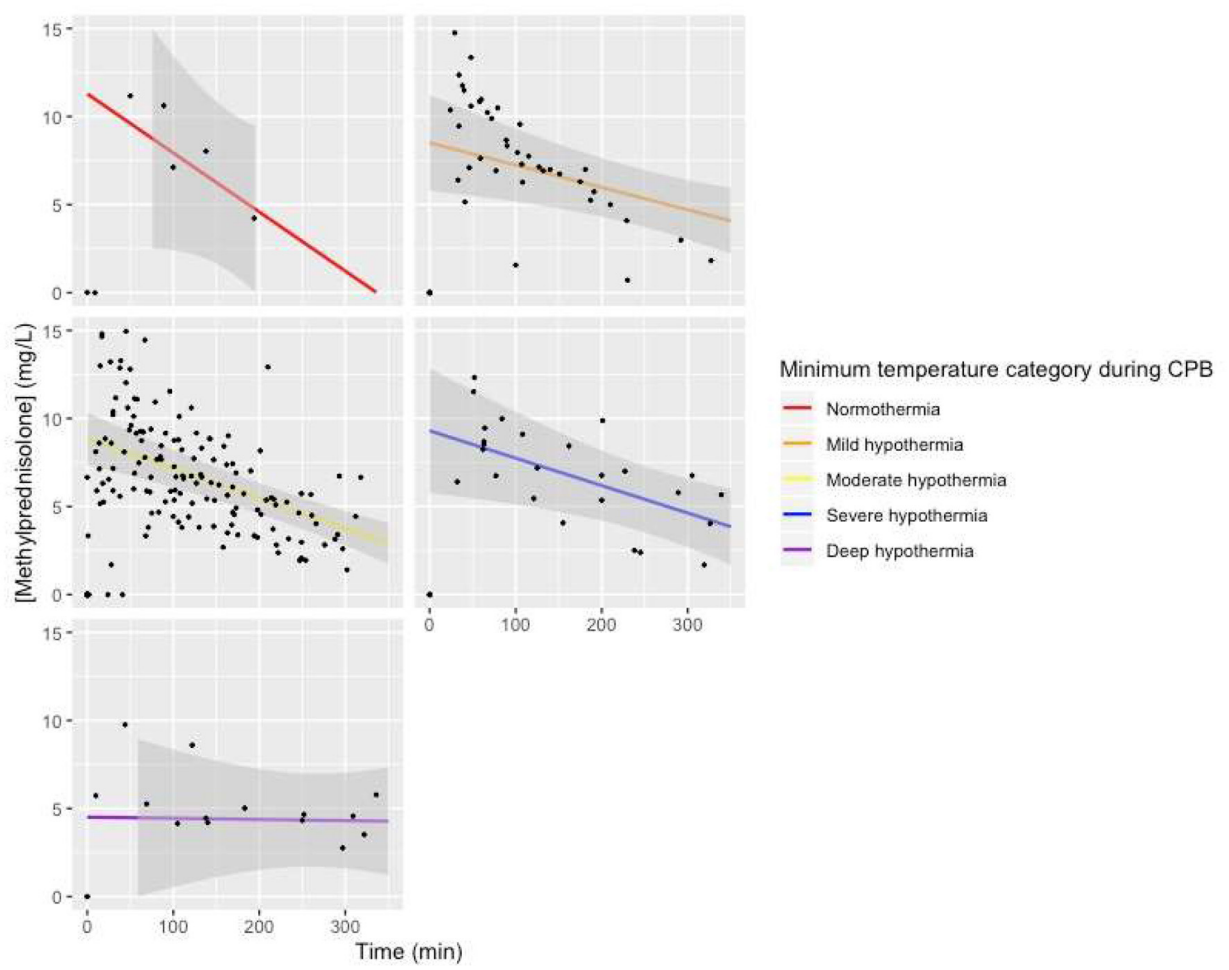
Predicted MP concentration (mg/L) vs. time (min) for temperature categories based on the linear mixed-effects model. Gray ribbon: 95% confidence interval. *T* = 0: start of CPB.

Visual representation of the model with minimum temperature during CPB added to the baseline model as a fixed effect with an interaction with time showed a lower intercept for patients with deep hypothermia, a less steep slope for patients with deep hypothermia, and a steeper slope for patients with normothermia, but with large confidence intervals (Figure 6).

Addition of diuresis to the baseline model as a categorical fixed effect [diuresis <4.7 ml/kg (25% quantile) vs. diuresis >21 ml/kg (75% quantile)] with an interaction with time showed no significant differences in intercept or slope (Supplementary Figure 12).

## Discussion

To our knowledge, this is the first publication of MP for congenital cardiac surgery incorporating data for children younger than 4 years old.

Even though MP is used regularly in pediatric cardiac surgery (20, 21), to our knowledge its pharmacokinetics and plasma concentrations have not been comprehensively investigated in children with congenital heart disease undergoing cardiac surgery with CPB. It is unknown whether there is a significant influence of CPB on the plasma concentrations of MP and whether this influence may be an explanation for the limited reported efficacy of steroid administration in cardiac surgery with CPB (10–12).

A 26.8% decrease in observed MP plasma concentration on initiation of CPB was shown in our study. For patients with additional CPB runs during one surgical procedure, the decrease in observed MP plasma concentration was shown only for the first initiation of CPB, because the same CPB system that was used on previous CPB runs was reused on ensuing runs in the same patient. Initiation of CPB causes the priming volume of the CPB system patient's so it becomes to be added to the patient's central compartment to be added to the central compartment, causing hemodilution. The observed percentage decrease in concentration of MP on initiation of CPB is smaller than what we would expect based on hemodilution alone, and we could not show a correlation with the volume of priming fluid. This smaller than expected decrease in observed concentration may be due to redistribution of MP from the child's peripheral tissues back to the central compartment. Unfortunately, no further information is available on the redistribution half-time of MP in children. Alternatively, MP may be formed from its prodrug MP hemisuccinate. The half-time of MP hemisuccinate is 20 to 30 min (13, 14). The amount of time that passed between the time of MP hemisuccinate administration to the time of measurement of the first MP plasma concentration after the start of CPB was 70 min (median, range = 35–151 min). Metabolism of MP hemisuccinate to MP is thus a less likely candidate to explain the smaller percentage decrease in concentration of MP on initiation of CPB, as most of MP hemisuccinate will have been metabolized to MP already.

Significant absorption of MP by plastic components of the CPB system is highly unlikely, given that the observed decrease in MP concentration on initiation of CPB is even smaller than the calculated concentration decrease based on hemodilution alone. This confirms the results of our previous *in vitro* study, which showed MP recoveries close to 100% for different CPB systems which were also used in the current study (22).

The decrease in observed concentration on initiation of CPB may in part be caused by metabolism of MP, as there is a time difference between the MP concentration measured before and after initiation of CPB of 13 min (median, range = 7–36 min). The observed MP concentration difference in our study was shown to be much larger than what can be explained by metabolism alone.

Studies performed in neonates (23) and infants (24) undergoing CPB and receiving an MP dose of 30 mg/kg showed similar concentrations to our study in similar patient groups. Thirty minutes after the initiation of CPB, neonates achieved MP plasma concentrations of 4 vs. 4.4 μg/ml (median, range = 3.8–7.7 μg/ml) in our study (23). Infants achieved MP plasma concentrations of 7.2 μg/ml 30 min after the initiation of CPB vs. 7.7 μg/ml (median, range = 6.27–10.6 μg/ml) in our study, and 6.2 μg/ml at protamine administration vs. 6.1 μg/ml (median, range = 1.82–9.9 μg/ml) in our study (24). Our study confirms the large interindividual variability in achieved MP plasma concentration with weight-based MP administration (13, 25).

The lower MP concentration achieved with the same 30-mg/kg dose in neonates that has been shown in our study was previously shown in the studies performed by Keski et al. (23–25). In our mixed-effects model-based concentration–time curve, we showed a significantly lower intercept for the MP plasma concentration for neonates (Figures 3A,B), without significant differences in the amount of time between administration of MP and initiation of CPB compared to other age groups. The lower estimated plasma concentration of MP at the beginning of CPB originated from before the initiation of CPB. These results suggest a larger volume of distribution (Vd) of MP in neonates, as Vd is calculated by dividing the amount of drug present in the body by administered dose. Developmental differences in Vd between different age groups have been described (4). Younger children have lower concentrations of binding proteins, larger circulating volumes, and a smaller percentage fat tissue.

Another covariate with a significantly lower intercept for MP in the model-based concentration–time curve was a large volume of cell saver blood processed during the procedure (Figures 5A,B). There were significant differences for various maturational covariates between groups with different volumes of cell saver blood processed; thus, we assume the lower intercept is based on an interaction with age group.

Patients who were treated for PHT with sildenafil showed a visual trend toward a lower intercept for MP in the model-based concentration–time curve (Figure 4). The possible influence of treatment of PHT with sildenafil on the Vd of MP thus needs to be investigated in further studies.

In a one-compartment model, drug only leaves the central compartment through elimination. The slope of the concentration–time curve of a drug is thus a representation of clearance (Cl). There was a significantly less steep slope of the model-based MP concentration–time curve in neonates compared to other age groups (*p* = 0.048), suggesting a lower Cl of MP for neonates than for other children. If we were to determine the half-life of MP based on the model-based MP concentration–time curve for different age groups, it would amount to longer than 6 h for neonates, 4.7 h for infants, 3.6 h for preschool children, and 4.7 h for school children. Developmental changes in Cl have been shown. Metabolism of cytochrome P450 (CYP) enzymes approaches approximately 50 to 70% of adult levels at birth. By 2 to 3 years, CYP activity is larger than adult values for selected isoenzymes, and by puberty, CYP activity decreases to adult levels (26). A previous study in children aged 4 to 15 years receiving MP for an array of autoimmune diseases showed large individual differences in Cl ranging from 0.18 to 0.93 L/kg per hour, with no correlation of Cl with age in the previously mentioned study, although patients with Cl larger than 0.5 L/kg per hour tended to be younger (13). We believe the conflicting results from this study as opposed to ours lie in the fact that the children in that study were older than those in our study, with metabolism already largely at mature levels. Comparison of the value of half-life for school aged children of 4.7 h in our study to the half-life of 2.5 h reported in a previous pediatric study (13) suggests that there is a similar decrease in Cl of MP in our study.

A large volume of cell saver blood processed was also associated with a less steep slope of the model predicted MP concentration vs. time curve (Figures 5A,B). We suspect that this covariate is a proxy covariate for a large group of other covariates, including patient characteristics and surgery-related factors.

Our study does not rule out an effect of temperature management on Cl. The MP plasma concentration vs. time curve was almost horizontal for one patient treated with DHCA and showed the steepest curve in one patient treated with normothermic CPB. Both categories contained only one patient; thus, it is pivotal that this covariate is investigated further in larger studies.

To our surprise, there is no information on the pharmacodynamics of MP with regard to the inflammatory response caused by CPB. It is thus unknown which “therapeutic levels” to aim for.

A randomized trial to determine the pharmacokinetics, pharmacodynamics, safety, and efficacy in infants undergoing heart surgery with CPB is, in our opinion, long overdue, with and is fortunately currently recruiting 1,200 patients (STRESS, https://clinicaltrials.gov/ct2/show/NCT03229538). In this study, MP 30 mg/kg at the time of initiation of CPB will be compared to placebo. The study will be completed beginning of 2021. Thirty milligrams per kilogram is still the most commonly administered dose of MP in pediatric cardiac surgery; thus, we understand why this dose has been chosen by the researchers. Comparable decreases in anti-inflammatory mediator concentration and clinical outcomes have been reported with MP doses of 5 mg/kg or even 2 mg/kg, however (25, 27).

## Limitations

Our study has several limitations. First, the number of patients in our study was relatively small.

We measured total plasma MP concentration and have no information on albumin levels before, during, or after CPB in our patients. Protein binding of MP is 78% (18), so a large change in unbound plasma concentrations of MP is not expected.

As the study was part of a larger PK study with the aim of performing population PK modeling for drugs commonly used during pediatric cardiac surgery requiring CPB, blood samples were taken at random timepoints. Usually, only a limited number of observations can be obtained in pediatric subjects; the population approach to obtain PK parameters is the preferred approach. The population approach allows for the analysis of sparse (limited number of observations per individual) and unbalanced data (unequal distribution of observations in various parts of the concentration–time profile in the individuals) or a combination of both. As a result of this methodology, when designing a pediatric study of which the data will be analyzed using the population approach, it is advisable to collect samples at different times (or time windows) in subgroups of patients (28).

Last, there were significant differences between subgroups. Since we performed an observational study, homogeneity of groups was not intended in the study. Population PK modeling has the goal of explaining interindividual differences.

## Conclusion

We found a significant decrease in MP plasma concentrations on initiation of CPB. Significant covariates of influence on the MP plasma concentration vs. time curve after initiation were age group (especially neonates) and large volume of cell saver blood processed. As there is no further information regarding pharmacodynamics of MP, we cannot currently recommend increasing the dose for neonates. We recommend further evaluation of the influence of treatment for PHT with sildenafil and temperature management.

## Data Availability Statement

The raw data supporting the conclusions of this article will be made available by the authors, without undue reservation.

## Ethics Statement

The studies involving human participants were reviewed and approved by Erasmus Medical Center Medical Ethical Review Board. Written informed consent to participate in this study was provided by the participants' legal guardian/next of kin.

## Author Contributions

AS and DT: study conception and design. AS and GZ-R data collection. AS, KV, and FS: analysis and interpretation of results. AS: draft manuscript preparation. GZ-R, KV, SW, RS, AB, and DT: revision manuscript. All authors reviewed the results and approved the final version of the manuscript.

## Conflict of Interest

The authors declare that the research was conducted in the absence of any commercial or financial relationships that could be construed as a potential conflict of interest.

## Publisher's Note

All claims expressed in this article are solely those of the authors and do not necessarily represent those of their affiliated organizations, or those of the publisher, the editors and the reviewers. Any product that may be evaluated in this article, or claim that may be made by its manufacturer, is not guaranteed or endorsed by the publisher.
