# Supplementary material for: Methylprednisolone Plasma Concentrations During Cardiac Surgery With Cardiopulmonary Bypass in Pediatric Patients
Source: Front Cardiovasc Med. 2021 Aug 25;8:640543. doi: 10.3389/fcvm.2021.640543 (PMC8424008; doi:10.3389/fcvm.2021.640543)
Supplement: Supplementary file 1 [file Data_Sheet_1.docx]

**SUPPLEMENTARY MATERIALS**

Fig S1 Predicted MP concentration (mg/l) versus time (min) for SpO2 based on the linear mixed effects model.


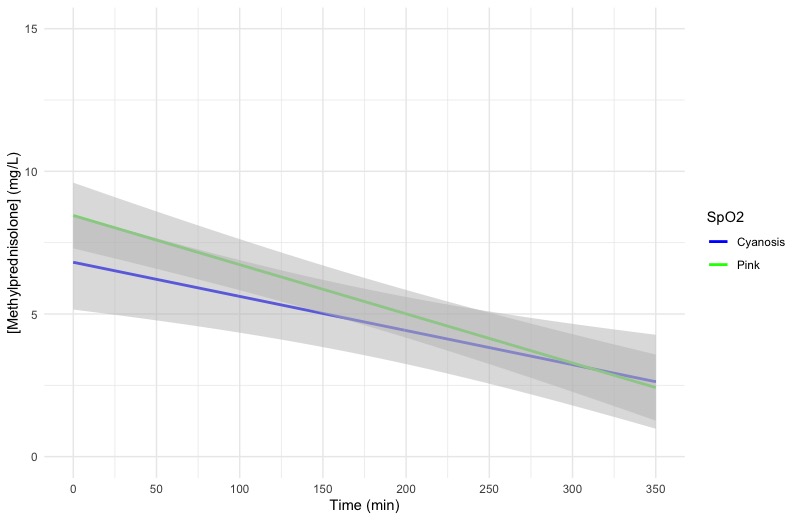


Grey ribbon: 95 % confidence interval

T = 0: start of CPB

Fig S2 Predicted MP concentration (mg/l) versus time (min) for all STSEACTS mortality risk categories based on the linear mixed effects model.


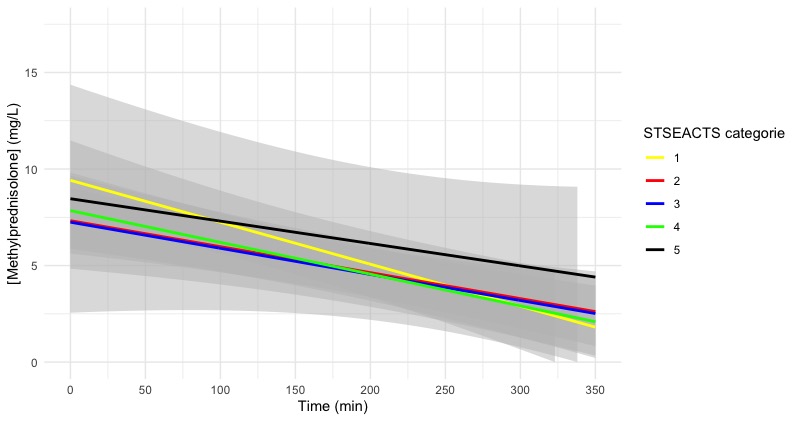


Grey ribbon: 95 % confidence interval

T = 0: start of CPB

Fig S3 Predicted MP concentration (mg/l) versus time (min) for sex based on the linear mixed effects model.


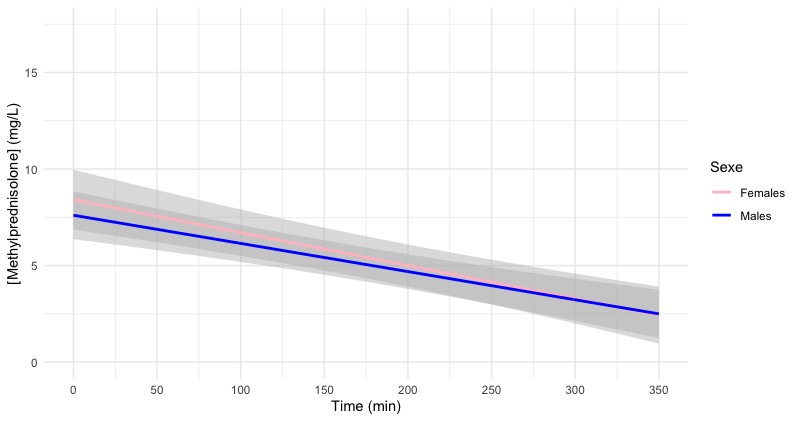


Grey ribbon: 95 % confidence interval

T = 0: start of CPB

Fig S4 Predicted MP concentration (mg/l) versus time (min) for volume overload based on the linear mixed effects model.


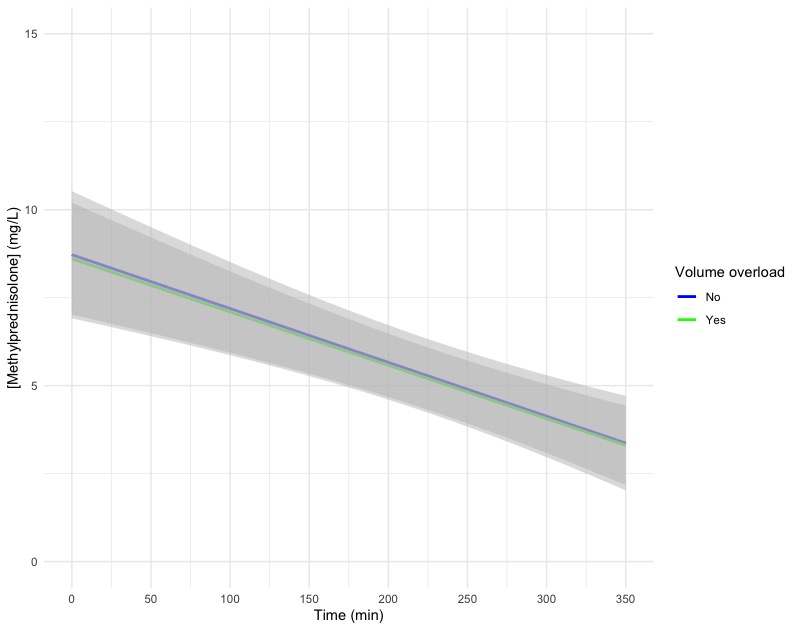


Grey ribbon: 95 % confidence interval

T = 0: start of CPB

Fig S5 Predicted MP concentration (mg/l) versus time (min) for roller pump vs centrifugal pump based on the linear mixed effects model.


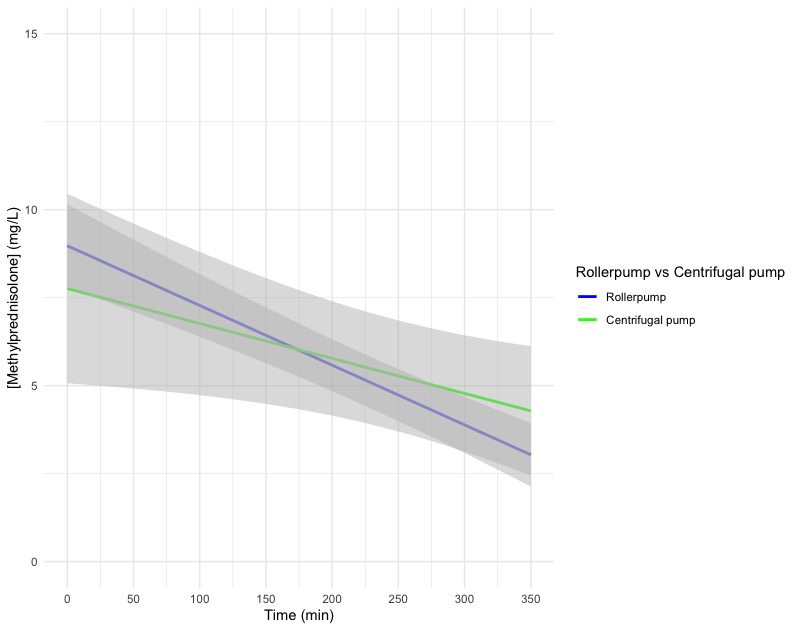


Grey ribbon: 95 % confidence interval

T = 0: start of CPB

Fig S6 Predicted MP concentration (mg/l) versus time (min) for FFP in the priming fluid based on the linear mixed effects model.


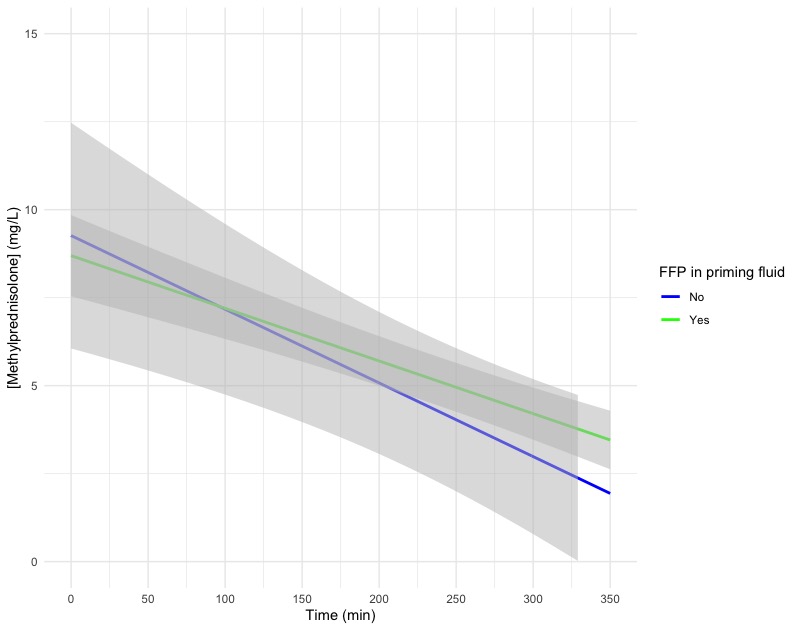


Grey ribbon: 95 % confidence interval

T = 0: start of CPB

Fig S7 Predicted MP concentration (mg/l) versus time (min) for PC in the priming fluid based on the linear mixed effects model.


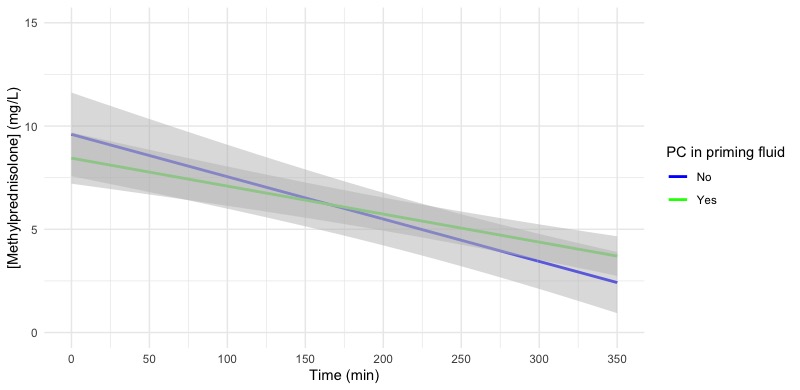


Grey ribbon: 95 % confidence interval

T = 0: start of CPB

Fig S8 Predicted MP concentration (mg/l) versus time (min) for aortic occlusion as a dichotomous variable based on the linear mixed effects model.


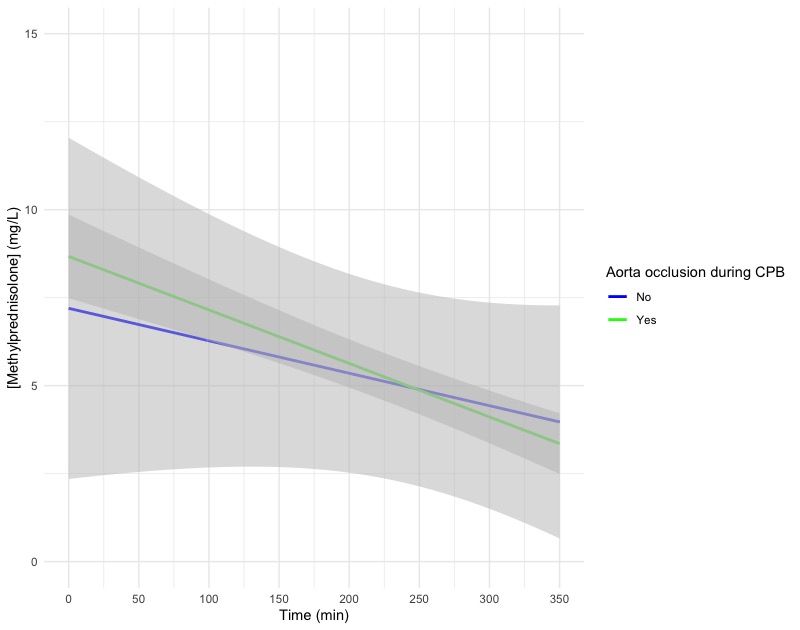


Grey ribbon: 95 % confidence interval

T = 0: start of CPB

Fig S9 Predicted MP concentration (mg/l) versus time (min) for multiple CPB runs as a dichotomous variable based on the linear mixed effects model.


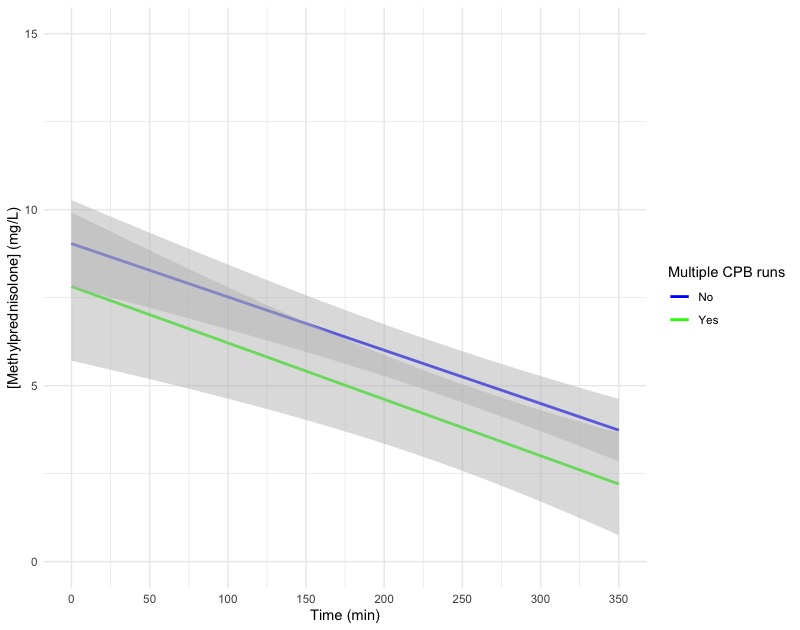


Grey ribbon: 95 % confidence interval

T = 0: start of CPB

Fig S10 Predicted MP concentration (mg/l) versus time (min) for use of a hemofilter as a dichotomous variable based on the linear mixed effects model.


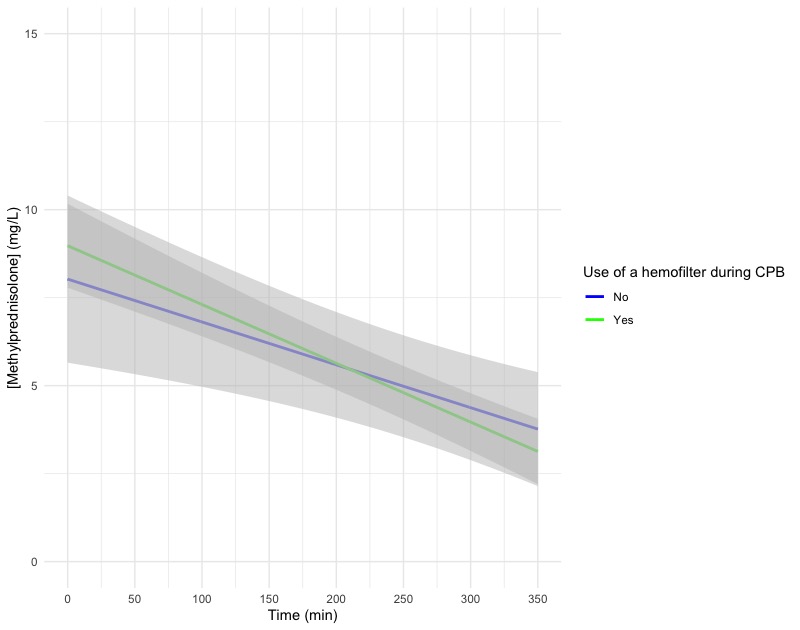


Grey ribbon: 95 % confidence interval

T = 0: start of CPB

Fig S11 Predicted MP concentration (mg/l) versus time (min) for perfusion time as a dichotomous variable based on the linear mixed effects model.


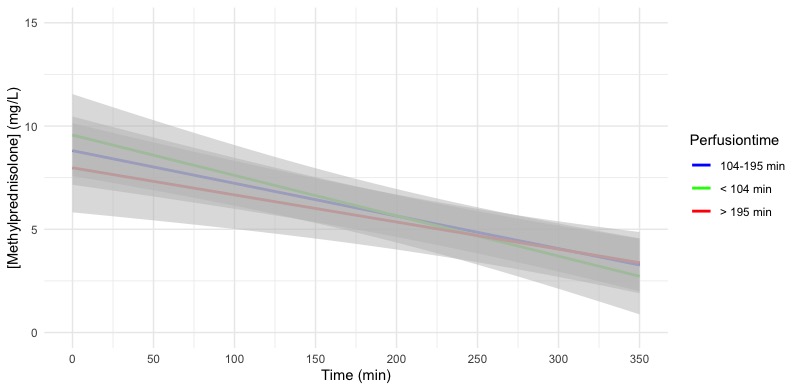


Grey ribbon: 95 % confidence interval

T = 0: start of CPB

Fig S12 Predicted MP concentration (mg/l) versus time (min) for diuresis as a categorical variable based on the linear mixed effects model.


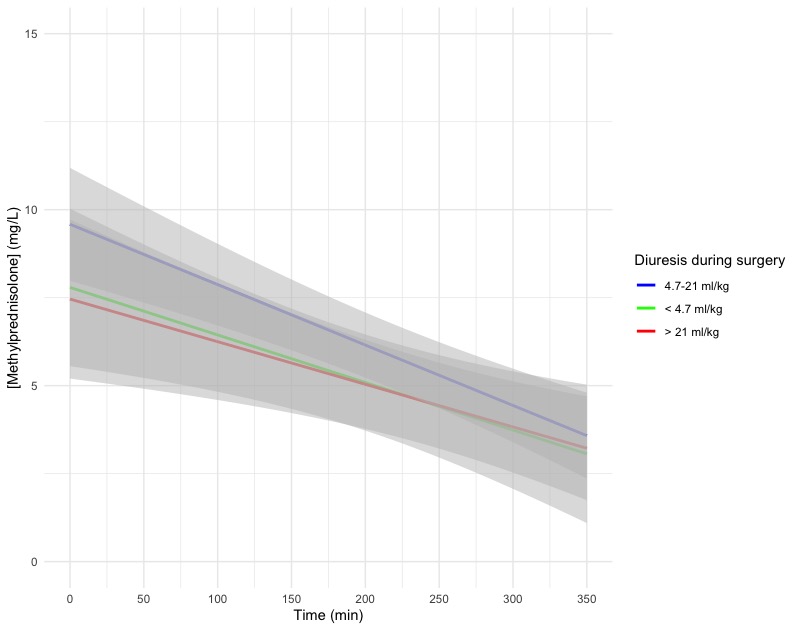


Grey ribbon: 95 % confidence interval

T = 0: start of CPB
